# Local treatment of a pleural mesothelioma tumor with ONCOS-102 induces a systemic antitumor CD8^+^ T-cell response, prominent infiltration of CD8^+^ lymphocytes and Th1 type polarization

**DOI:** 10.4161/21624011.2014.958937

**Published:** 2014-12-15

**Authors:** Tuuli Ranki, Timo Joensuu, Elke Jäger, Julia Karbach, Claudia Wahle, Kalevi Kairemo, Tuomo Alanko, Kaarina Partanen, Riku Turkki, Nina Linder, Johan Lundin, Ari Ristimäki, Matti Kankainen, Akseli Hemminki, Charlotta Backman, Kasper Dienel, Mikael von Euler, Elina Haavisto, Tiina Hakonen, Juuso Juhila, Magnus Jaderberg, Petri Priha, Lotta Vassilev, Antti Vuolanto, Sari Pesonen

**Affiliations:** 1 Oncos Therapeutics; Helsinki, Finland; 2 Docrates Cancer Center; Helsinki, Finland; 3 Onkologie-Hämatologie; Krankenhaus Nordwest; Frankfurt, Germany; 4 Institute for Molecular Medicine Finland (FIMM); Helsinki, Finland; 5 Division of Pathology; HUSLAB and Haartman Institute; Helsinki University Central Hospital; Helsinki, Finland; 6 Genome-Scale Biology; Research Programs unit; University of Helsinki; Helsinki, Finland; 7 Cancer Gene Therapy Group; Haartman Institute; University of Helsinki; Helsinki, Finland

**Keywords:** Adenovirus, antitumor immunity, cytotoxic immunotherapy, GM-CSF, Th1 polarization, tumor infiltrating lymphocytes, APC, antigen presenting cell, CCL2, (C-Cmotif) ligand 2, CTCAE, common terminology criteria for adverse events, CX3CL1, (C-X3-C motif) ligand 1, CXCL9, (C-X-C motif) ligand 9, CXCL10, (C-X-C motif) ligand 10, ELISPOT, enzyme-linked immunospot assay, GM-CSF, granulocyte macrophage colony stimulating factor, IFNg, interferon gamma, IRF1, interferon regulatory factor 1, PET, positron emission tomography, RANTES, regulated on activation, normal T cell expressed and secreted, TILs, tumor infiltrating lymphocytes, VP, viral particle

## Abstract

Late stage cancer is often associated with reduced immune recognition and a highly immunosuppressive tumor microenvironment. The presence of tumor infiltrating lymphocytes (TILs) and specific gene-signatures prior to treatment are linked to good prognosis, while the opposite is true for extensive immunosuppression. The use of adenoviruses as cancer vaccines is a form of active immunotherapy to initialise a tumor-specific immune response that targets the patient's unique tumor antigen repertoire. We report a case of a 68-year-old male with asbestos-related malignant pleural mesothelioma who was treated in a Phase I study with a granulocyte-macrophage colony‑stimulating factor (GM-CSF)-expressing oncolytic adenovirus, Ad5/3-D24-GMCSF (ONCOS-102). The treatment resulted in prominent infiltration of CD8^+^ lymphocytes to tumor, marked induction of systemic antitumor CD8^+^ T-cells and induction of Th1-type polarization in the tumor. These results indicate that ONCOS-102 treatment sensitizes tumors to other immunotherapies by inducing a T-cell positive phenotype to an initially T-cell negative tumor.

Mechanisms by which cancer cells die, shape the early stages of tumor associated antigen presentation and are essential for the elicitation of durable anticancer immune responses.^1,2^ Adenoviruses cause immunogenic cancer cell death associated with the release of natural adjuvants from within the dying cells,^3^ which may augment the recognition of tumor antigens previously hidden from the immune system, and result in subsequent priming of potent tumor-specific immunity.^4,5^ This effect may be further enhanced by immune-stimulating transgenes expressed by the virus. ONCOS-102 is a serotype 5 adenovirus that features a chimeric capsid for enhanced gene delivery to cancer cells and a 24 bp deletion in Rb binding site of E1A for cancer cell restricted replication, and is armed with GM-CSF, a potent inducer of antitumor immunity (6). GM-CSF functions by directly recruiting antigen presenting cells (APC) and natural killer cells, as well as by activating and maturing APCs at the tumor site.^7,8^

Malignant pleural mesothelioma is a rare but aggressive cancer with increasing incidence associated with asbestos exposure.^9^ Most mesotheliomas originate in the pleura and more than 80% of patients with pleural mesothelioma are men. Patients with malignant pleural mesothelioma have a poor prognosis with an estimated median survival time varying from 4 to 12 mo post-diagnosis.^10^

The 68-year-old patient with malignant pleural mesothelioma had previously been treated with two chemotherapy regimens (cisplatin + pemetrexed, docetaxel alone) and radiotherapy, but despite treatment, the disease continued to progress. 16 mo after diagnosis the patient was treated in a Phase I study (NCT01598129) with ONCOS-102. The Phase I study was approved by the Ethics Committee in Finland.

Adenoviruses possess a unique ability to prime and boost immune responses.^11^ The patient was treated with four closely timed intratumoral injections of 3 × 10^11^ viral particles (VP) of ONCOS-102 on days 1, 4, 8 and 15. Frequent dosing was used to elicit efficient viral replication and cancer cell lysis for priming of robust immune response, and further injections were given on days 29, 57, 85, 113 and 141 to boost the therapeutic effects. For downregulation of immunosuppressive regulatory T-cells, a daily dose of 50 mg/d oral cyclophosphamide was included.

According to common terminology criteria for adverse events (CTCAE), treatment resulted in grade 1 and 2 adverse events with the exception of grade 3 fever. Treatment-related innate immune response manifested by immediate short-term increase of pro-inflammatory cytokines IL-6 and IL-8 was seen in serum after each viral injection.

The presence of TILs, especially CD8^+^ T-cells, has been recognized as a marker of antitumor immune response across a wide range of tumors.^12-16^ Markedly, a positive correlation has been linked to high TIL counts at pre-treatment samples and good prognosis.^17^ We observed a prominent post-treatment increase in the number of TILs by immunohistochemical staining in biopsy 29 d after treatment initiation compared to baseline. Importantly, a 131-fold increase in CD8^+^ T-cells was seen in post-treatment (**Fig. 1**), while the pre-treatment sample had very few infiltrating CD8^+^ T-cells, suggesting that treatment with ONCOS-102 induced a robust CD8^+^ T-cell response.

**Figure 1.**
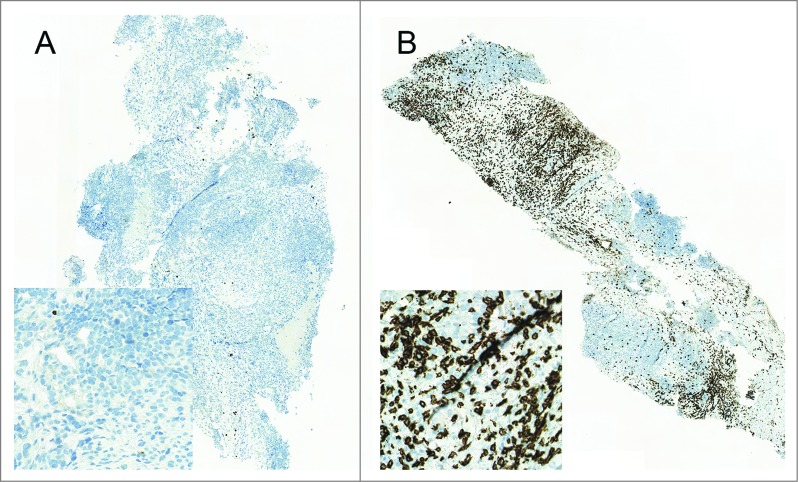
Infiltration of CD8^+^ T-cells to tumor. Biopsies were obtained at baseline (**A**) and 29 d after the treatment initiation (**B**) and stained for CD8^+^ T-cells. Briefly, three micron sections were cut from formalin-fixed and paraffin-embedded tissues and processed for immunohistochemistry performed with Ventana BenchMark XT immunostainer (Ventana Medical Systems, Tucson, AZ USA) using CD8^+^ rabbit monoclonal antibody (clone SP57, ready to use, Ventana, Roche) and visualized using UltraViewDabv3 with amplification (Ventana). The specimens were counterstained with hematoxylin and post counterstained with bluing reagent. Positive staining is shown in brown.

Central and peripheral tolerance has led to the relative paucity of tumor antigen-specific T-cells as compared to antiviral T-cells, leading to a situation where the immune response may be dominated by the more numerous and higher quality antiviral T-cells over antitumor T-cells.^18^ To analyze whether treatment with ONCOS-102 elicited antitumor CD8^+^ T-cell response, IFNγ enzyme linked immunospot assay (ELISPOT) was performed from pre- and post-treatment samples. A prominent post-treatment induction of MAGE-A3-specific (peptide p271–279 FLWGPRALV) CD8^+^ T-cells in PBMCs was seen 29 d after treatment initiation suggesting that intratumoral treatment with ONCOS-102 elicits systemic tumor specific immunity despite the presence of viral antigens (**Fig. 2**).

**Figure 2.**
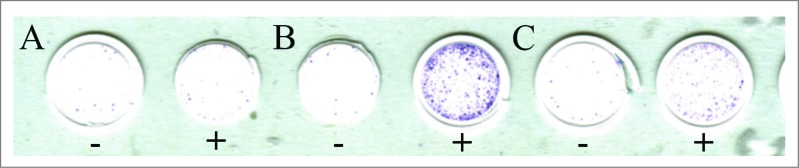
IFNγ enzyme linked immunospot assay (ELISPOT) for MAGE-A3-specific (p271–279 FLWGPRALV) CD8^+^ T-cells was performed. Purified CD8^+^ were pre-sensitized with peptide-pulsed, irradiated autologous PBMCs depleted of CD4^+^ and CD8^+^ T-cells and tested on day 10 by IFNγ ELISPOT assay for recognition of autologous antigen-presenting cells. The number of cytokine-producing antigen-specific T-cells was evaluated using AID EliSpot Reader Classic ELR 07 (Autoimmun Diagnostika GmbH, Strassberg, Germany). Cells without peptide stimulation (−) and with MAGE-A3 stimulation (+) are shown from samples obtained at baseline (**A**), 1–4 weeks after treatment initiation (**B**) and 20–24 weeks after treatment initiation (**C**).

TILs in an established progressing cancer often show an exhausted functional state with impaired effector cytokine production,^19^ which is similar to chronic viral infection due to persistent tumor-antigen load and immunosuppressive factors in the tumor microenvironment.^20^ Microarray analysis of tumor biopsies showed markedly elevated expression levels of genes encoding cytotoxic factors perforin, granzyme B and granulysin post-treatment (**Fig. 3**), suggesting that the treatment-induced TILs displayed effector functionality. Further, elevated expression levels of genes encoding Th1 associated factors interferon gamma (IFNγ) and interferon regulatory factor 1 (IRF1), and Th1 associated chemokines (CCL2, RANTES, CX3CL1, CXCL9 and CXCL10) were seen post-treatment as well. Upregulation of these genes has previously been associated with Th1 type adaptive immunity and has been proposed to form a major component of a gene-signature predictive for good prognosis in colon cancer patients.^21,22^ Altogether, these results indicate that treatment with ONCOS-102 elicits tumor specific T-cell responses and induces tumor infiltration of cytotoxic T-cells with effector functions.

**Figure 3.**
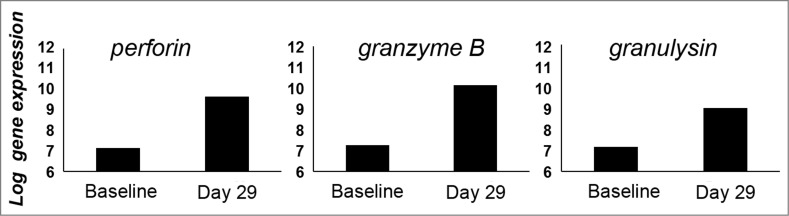
Total RNA was extracted from snap-frozen core needle tumor biopsies taken at baseline and 1 mo after the treatment initiation and gene expression profiling was performed by using HumanHT-12 Illumina microbead chips (Illumina Inc., San Diego, CA USA). The average signal values were quantile-normalized, log2-transformed, and annotated using the package lumi (Bioconductor open source software). Chip-dependent batch-effects were removed using empirical Bayes methods. Markedly elevated expression levels of genes encoding cytotoxic factors perforin, granulysin and granzyme B in post-treatment sample suggest that CD8^+^ TILs show an effector phenotype.

While the initial immune response to a single antigen is quite brisk once the antigen is recognized as non-self, the activated immune response may in turn lead to the development of delayed yet long-lived memory response that can sustain clinical benefit beyond the period of treatment.^23^ A late decrease in metabolic activity was observed in positron emission tomography (PET) imaging 7.5 mo after treatment initiation during the follow-up period after the end of trial, measured as a 47% decrease in total lesion glycolysis in comparison to the previous imaging at the 6 mo time point (**Fig. 4**). The patient had not received additional treatments after the trial before the last imaging, suggesting that the tumor-specific immune response elicited by ONCOS-102-treatment was robust enough to partially eradicate the tumor load. This patient survived 18 mo (542 d) from the treatment initiation and over 33 mo (999 d) from diagnosis, which is remarkable given that the median survival of patients with malignant pleural mesothelioma varies from 4 to 12 mo from diagnosis.

**Figure 4.**
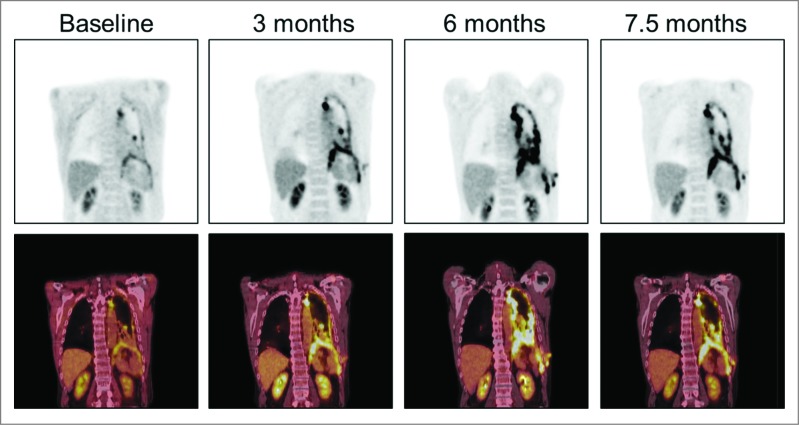
PET images at baseline and 3 , 6 and 7.5 mo after treatment initiation. A 47% decrease in the metabolic activity as measured by total lesion glycolysis in positron emission tomography in comparison to the previous imaging at the 6 mo time point was seen at the 7.5 mo time point.

In summary, local treatment with ONCOS-102 induces dense infiltration of CD8^+^ T-cells to tumor and elicits systemic tumor-specific CD8^+^ T-cell responses, despite the presence of a high load of viral antigens in the tumor microenvironment. Th1 polarization in tumors is related to a better prognosis in various cancer types.^12-16^ Recent findings indicate that poorly immunogenic tumors with no infiltrating T-cells are not responsive to checkpoint inhibitors.^24^ The potential ability of ONCOS-102 to sensitize tumors to other immunotherapies by inducing CD8^+^ T-cell responses in T-cell negative tumors encourages investigating the use of ONCOS-102 in combination with other immunotherapies.
